# Chemical composition and physical characteristics of faeces in horses with and without free faecal liquid – two case-control studies

**DOI:** 10.1186/s12917-021-03096-1

**Published:** 2022-01-03

**Authors:** K. M. Lindroth, J. Dicksved, I. Vervuert, C. E. Müller

**Affiliations:** 1 Department of Animal Nutrition and Management, Swedish University of Agricultural Sciences, 750 07 Uppsala, Sweden; 2 Institute of Animal Nutrition, Nutrition Diseases and Dietetics, Faculty of Veterinary Medicine, University of Leipzig, D-04159 Leipzig, Germany

**Keywords:** Equine, Free faecal water, Lactic acid, Particle size, SCFA, Water absorptive capacity

## Abstract

**Background:**

Free faecal liquid (FFL) is a condition in horses characterised by two-phase (one solid and one liquid) separation of faeces. Causes of the condition are unknown, but disturbed hindgut fermentation has been suggested as it may alter biochemical composition and appearance of faeces in equines. However, information on faecal composition in horses with FFL is scarce. Faecal chemical composition (dry matter, osmolality, ash, macro minerals, short-chain fatty acids (SCFA) and pH) and physical characteristics (free liquid, sand, water holding capacity and particle size distribution) were compared in horses with (case) and without (control) FFL in two sub-studies. In sub-study I, faeces from 50 case-control horse pairs in Sweden and Norway were sampled in three sampling periods (SP1-SP3). In sub-study II, faeces from 32 case-control horse pairs in Germany were sampled on one occasion.

**Results:**

In sub-study I, faecal concentration and proportion of lactic acid (of total short-chain fatty acids, SCFA) and water holding capacity was lower in case compared to control horses. Other variables (content of dry matter, ash, sodium, calcium, phosphorous, magnesium, sulphur, and concentrations of *i*-butyric, *n*-valeric and total SCFA, ammonia-N as proportion of total N, and pH) were similar in faeces from case and control horses. In sub-study II, all analysed variables were similar in faecal samples from case and control horses. Faecal particle size distribution was similar in case and control horses, but the proportion of larger particles (2 and 1 mm) were lower and proportion of smaller particles (< 1 mm) was higher in sub-study I compared to in sub-study II.

**Conclusions:**

To the authors’ knowledge, this is the first study to investigate faecal chemical composition and physical characteristics in horses with FFL. Case and control horses had similar total SCFA, pH and osmolality, indicating that hindgut fermentation was similar. However, small differences in concentration and proportion (of total SCFA) of lactic acid and water holding capacity of faeces were shown and are of interest for further studies of horses with FFL.

## Background

Free faecal liquid (FFL), also referred to as faecal water syndrome (FWS), in horses manifests as differential solid and liquid phases at defecation, where the liquid phase is voided before, during, after or separately from defecation of the solid phase [1, 2]. The condition may last from a few days to months and sometimes years, and it may vary in severity and/or continuity over time [3, 4]. Causes of the condition have not been identified, but both intrinsic horse-related factors (e.g. low rank in the social hierarchy, being of paint-colour and being a gelding) and environmental factors (e.g. changes in management and type and amount of feeds) have been suggested to be important [1–4].

Information on faecal composition in horses with FFL is scarce, but could provide clues on the aetiology of this condition. Variables commonly used to describe digestion and hindgut disturbances in horses through analysis of faecal samples include pH [5–7], osmolality and concentration of individual and total volatile fatty acids (VFA) [8], particle size distribution [9–11] and presence of sand [12] and macro minerals [13]. Low faecal pH (< 6), together with high lactic acid concentration, has been used as an indicator of hindgut acidosis when e.g. starch-rich diets are fed to horses [5–7], while high faecal pH (> 7) has been reported in horses with osmotic diarrhoea [14]. Higher proportion of faecal *i*-butyrate to total VFA has been reported in diarrhoeic horses compared to healthy controls [8]. Particle size distribution in faeces has been used as an indicator of the function of mastication in equines [11] and horses with large colon impaction were found to have smaller faecal particle size than controls [9]. Presence of sand in equine faeces has been associated with sand accumulation in the hindgut, which may result in diarrhoea and impaction colic [12]. Faecal content of macro minerals has been reported to be higher in horses with diarrhoea compared to control horses [13, 14]. These, or other variables describing the chemical composition and physical characteristics of faeces has not been evaluated in horses with FFL. Therefore, the aim of this study was to compare chemical composition and physical characteristics of faeces in horses with and without FFL, in order to identify factors of potential importance in the aetiology of FFL.

## Results

### Sub-study I (Sweden-Norway)

#### Faecal chemical composition and physical characteristics

**Fig. 1:**
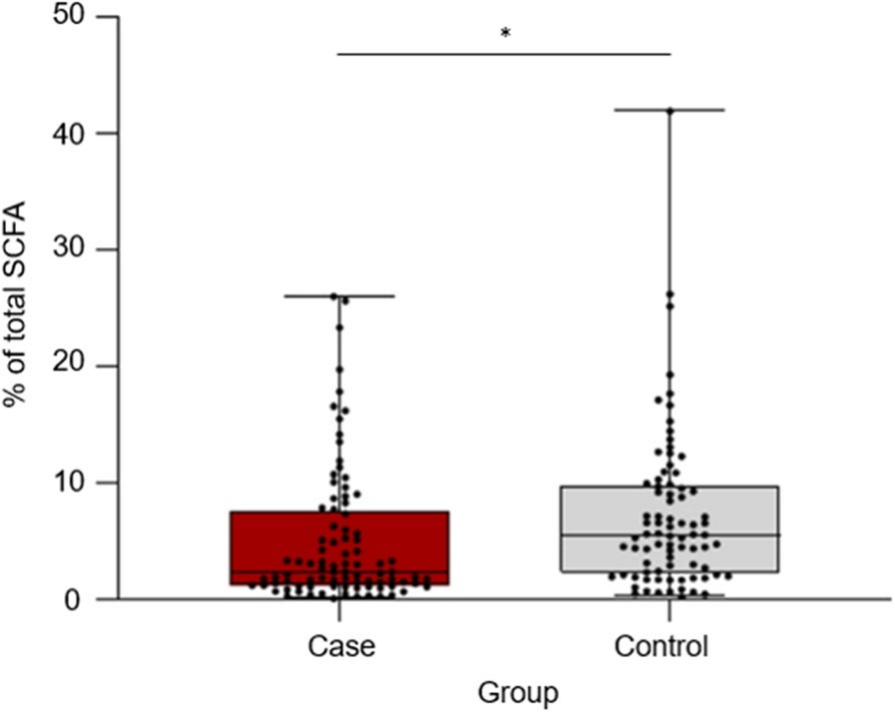
Lactic acid as percentage of total SCFA in faecal samples from Swedish and Norwegian horses with (case) and without (control) FFL (FFL) (sub-study I). *p < 0.05. Boxplots illustrate median (line in centre of box) and interquartile range of data (25th to 75th percentile). Whiskers represent the variability outside the upper and lower percentiles. Dots above boxplots show outliers

Concentration (*p* = 0.04, Table 1) and proportion (p = 0.04, Fig. 1) of lactic acid in total short-chain fatty acids (SCFA) were lower in case compared with control horses in sub-study I. For the other SCFAs, the concentration and proportion of total SCFA were similar between case and control horses (*p* > 0.05) (Table 1). Case and control horses had similar content of faecal dry matter (DM), ash, Na, Ca, P, Mg, K, S, ammonia-nitrogen (NH3-N), pH and ratio between sum of acetic- and butyric acid to propionic acid (C2 + C4/C3) (*p* > 0.05) (Table 1). No interaction effects between case/control and sampling period (SP) or differences due to SP were found for any of the faecal chemical components (*p* > 0.05).

**Table 1:** Chemical composition in faecal samples from horses with (case) and without (control) free faecal liquid (FFL) in Sweden and Norway (sub-study I)

	Case		Control		*P-value*
	Mean	SD	Mean	SD	
DM, g/kg	184.2	10.50	185.3	9.43	0.78
Ash, g/ kg DM	86.0	27.23	81.3	27.45	0.30
Sodium, g/ kg DM	2.2	2.09	2.1	1.79	0.25
Potassium, g/ kg DM	12.6	3.83	10.9	3.64	0.33
Calcium, g/ kg DM	4.3	1.57	4.1	1.60	0.68
Phosphorous, g/ kg DM	4.3	1.19	4.6	1.28	0.65
Magnesium, g/ kg DM	2.2	0.90	2.3	0.90	0.52
Sulphur, g/ kg DM	1.6	0.32	1.6	0.30	0.82
Ammonia-N, ml/g	119.1	70.13	126.5	80.12	0.67
Osmolality, osmol/kg	149.4	43.63	145.1	44.01	0.92
pH (log)	6.60	0.342	6.52	0.325	0.70
Lactic acid, mmol/l	1.9	2.42	2.3	2.15	0.04
Acetic acid, mmol/l	25.7	12.85	23.0	13.66	0.43
Propionic acid, mmol/l	7.9	4.00	7.3	3.99	0.56
*i*-Butyric acid, mmol/l	0.9	0.36	0.8	0.36	0.29
*n*-Butyric acid, mmol/l	2.4	1.66	1.9	1.54	0.34
*i*-Valeric acid, mmol/l	0.7	0.46	0.7	0.43	0.46
*n*-Valeric acid, mmol/l	0.5	0.19	0.5	0.20	0.98
Total SCFA, acid, mmol/l	41.6	19.64	39.0	19.84	0.49
C2 + C4/C3^a^	3.8	1.21	3.4	1.11	0.21
Lactic acid, % of SCFA	5.1	5.89	7.2	6.71	0.04
Acetic acid, % of SCFA	63.8	8.3	60.6	10.59	0.20
Propionic acid, % of SCFA	21.1	5.39	22.6	7.45	0.55
*i*-Butyric acid, % of SCFA	2.5	1.33	2.6	1.66	0.77
*n*-Butyric acid, % of SCFA	5.3	2.32	4.6	2.33	0.16
*i*-Valeric acid, % of SCFA	1.8	1.27	2.1	1.49	0.70
*n*-Valeric acid, % of SCFA	0.9	0.24	0.9	0.22	0.06
Sand, mm	0.1	0.13	0.0	0.04	0.05
Free liquid after centrifugation, ml	3.8	2.43	3.0	2.22	0.14

^a^Ratio between acetic acid (C2), propionic acid (C3) and butyric acid (C4)

**Fig. 2:**
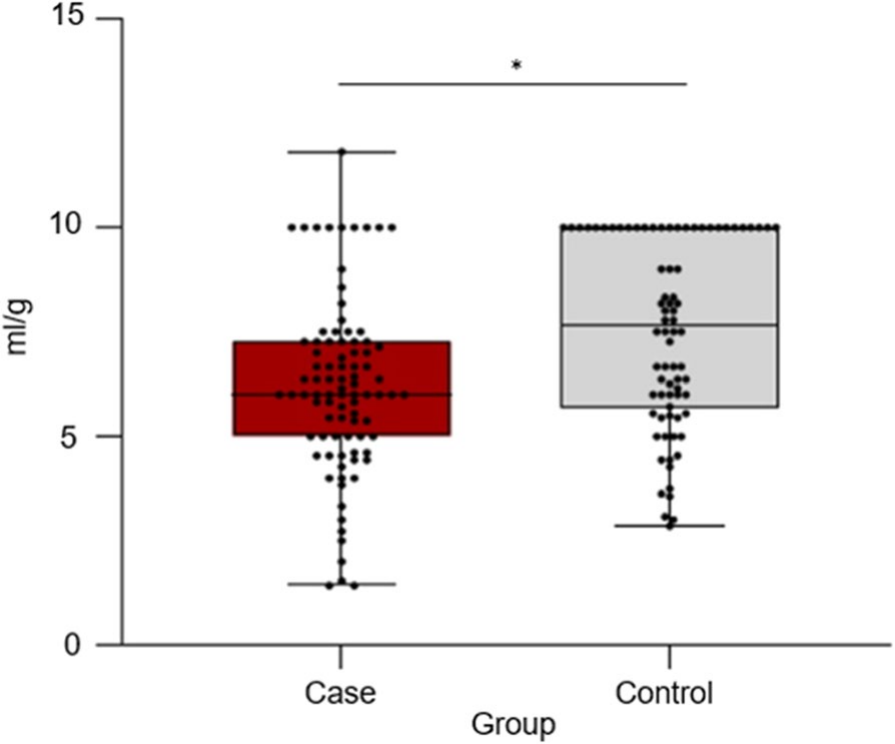
Water holding capacity (mL water per g dried faeces) in faeces from Swedish and Norwegian horses with (case) and without (control) FFL (sub-study I). *p ≤ 0.05. Boxplots illustrate median (line in centre of box) and interquartile range of data (25th to 75th percentile). Whiskers represent the variability outside the upper and lower percentiles. Dots above boxplots show outliers

Faeces from case horses had lower water holding capacity than faeces from control horses (*p* = 0.03) (Fig. 2). However, similar volumes of free liquid and sand were observed for case and control horses (*p* > 0.05) (Table 1). No interaction effects between case/control and SP or general differences between SPs were present for any of the measured variables.

### Sub-study II (Germany)

#### Faecal chemical composition

Concentration and proportion of individual SCFAs to total SCFA did not differ between case and control horses (*p* > 0.05) in sub-study II (Table 2). Case and control horses had similar content of DM, ash, K, Mg in their faeces and similar faecal pH and C2 + C4/C3 ratio (*p* > 0.05) (Table 2).

**Table 2:** Faecal chemical composition in horses with (case) and without (control) FFL in Germany (sub-study II)

Chemical composition	Case		Control		*P-value*
	Mean	SD	Mean	SD	
DM, g/kg	213.1	25.76	217.7	34.56	0.55
pH (log)	6.78	0.370	6.84	0.302	0.44
Ash, g/kg DM	9.4	3.36	8.9	3.72	0.49
Potassium, g/kg DM	9.2	2.43	9.6	3.71	0.50
Magnesium, g/kg DM	2.0	0.56	1.8	0.47	0.30
*L*-lactic acid, mmol/l	0.04	0.031	0.06	0.041	0.17
*D*-lactic acid, mmol/l	0.03	0.019	0.04	0.024	0.15
Acetic acid, mmol/l	15.5	7.94	17.1	9.14	0.62
Propionic acid, mmol/l	4.9	3.29	5.7	5.02	0.63
*i*-Butyric acid, mmol/l	0.6	0.47	0.8	0.98	0.45
*n*-Butyric acid, mmol/l	2.1	1.77	2.5	2.95	0.65
*i*-Valeric acid, mmol/l	0.6	0.53	0.7	0.73	0.79
*n*-Valeric acid, mmol/l	0.7	0.69	0.7	0.98	0.91
n-Caproic acid, mmol/l	0.5	0.50	0.5	0.71	0.32
Total SCFA, mmol/l	25.0	14.19	28.1	19.71	0.66
C2 + C4/C3^1^	3.8	0.84	4.0	0.96	0.31
Lactic acid, % of SCFA	0.4	0.33	0.5	0.38	0.30
Acetic acid, % of SCFA	64.4	8.39	65.4	8.18	0.90
Propionic acid, % of SCFA	19.1	2.68	18.9	3.13	0.41
*i*-Butyric acid, % of SCFA	2.4	0.62	2.3	0.86	0.60
*n*-Butyric acid, % of SCFA	7.6	3.28	7.3	2.80	0.56
*i*-Valeric acid, % of SCFA	2.4	0.91	2.2	0.74	0.46
*n*-Valeric acid, % of SCFA	2.3	1.44	1.9	1.17	0.38

### Faecal particle size distribution in sub-studies I and II (Sweden-Norway and Germany)

Case and control horses had similar faecal particle size distribution (*p* > 0.05) (Table 3). More than half of the sample (proportion of dry weight) consisted of particles < 1.0 mm for both case and control horses (Table 3). Differences were found between the sub-studies, as horses stabled in Germany had higher proportions of particles on sieve mesh sizes 2.0 (*p* = 0.005) and 1.0 mm (*p* < 0.0001) and a lower proportion of particles < 1.0 mm (*p* < 0.0001) compared with horses stabled in Sweden and Norway (Table 3).

**Table 3:** Faecal particle size distribution (%, on DM basis) in horses with (case) and without (control) FFL in Sweden and Norway (sub-study I), and Germany (sub-study II) for sieve mesh sizes 8, 4, 2, 1 and <  1 mm. Average and standard deviation (SD)

Group			Sieve mesh size				
			8 mm	4 mm	2 mm	1 mm	< 1 mm
Case/ Control	Case	Mean	4.0	10.3	20.9	11.5	53.4
		SD	2.93	4.95	5.98	8.18	14.50
	Control	Mean	3.6	11.0	22.0	11.4	52.1
		SD	1.78	5.38	6.23	7.85	15.43
Sub-study	Sub-study I	Mean	4.3	8.1	17.8	5.8	64.0
		SD	0.85	1.17	1.11	0.97	1.92
	Sub-study II	Mean	3.0	14.6	27.2	20.1	35.4
		SD	3.59	6.32	6.35	6.23	8.21
*p-value*	Case/ Control		0.83	0.41	0.30	0.95	0.18
	Sub-study		0.59	0.05	0.005	< 0.0001	< 0.0001

## Discussion

### Faecal chemical composition

Faecal lactic acid concentration and proportion of lactic acid to total SCFA were similar or lower in case compared to control horses in both sub-studies, which indicates that hindgut acidosis due to high lactic acid concentrations was not present in horses with FFL.

High concentrations or proportions of lactic acid in faeces has previously been associated with abrupt inclusion of starch-rich feeds in equine diets [15, 16], in horses fed concentrates and hay compared with hay only [17–19] and in horses with laminitis induced by creating hindgut acidosis [19–23]. In this study, horses were fed comparably low amounts of concentrates, especially in sub-study I, and hindgut acidosis due to high starch concentration in the diet was not likely in either case or control horses.

Horses with chronic diarrhoea have been reported to have lower faecal concentration of acetic acid, higher concentration of i-butyric acid, higher total VFA and higher proportion of i-butyric acid in total VFA in faeces compared with healthy horses [8]. None of these differences was found in the FFL horses in the present study. Similar faecal concentrations of acetic, butyric, propionic and valeric acid and total SCFA were found in case and control horses in both sub-studies. Also, similar proportions of individual SCFA to total SCFA and similar (C2 + C4)/C3) ratio were found in case and control horses in both sub-studies, indicating that FFL was not associated with disturbed hindgut fermentation in case horses.

Faecal pH was similar in case and control horses (> 6.0 and < 7) in both sub-studies. Low faecal pH (< 6.0) is often regarded as a sign of disturbed hindgut function, and has been observed in horses with hindgut acidosis resulting from a starch-rich diet [5, 6] or in horses with laminitis induced by large doses of non-structural carbohydrates [20–23]. High faecal pH (> 7) has been reported in horses with osmotic diarrhoea [14]. As faecal pH in the current study was higher than observed in horses with hindgut acidosis and lower than observed in horses with osmotic diarrhoea, these conditions were not considered to be present in case or control horses.

Faecal mineral content and osmolality were similar in case and control horses in the current study. Faecal content of macro minerals have previously been reported to be higher in horses with osmotic diarrhoea, usually expressed as watery diarrhoea [13, 14]. This indicates that FFL is most likely not due to increased osmolality in the hindgut and that it should not be considered osmotic diarrhoea. As faecal pH, osmolality and total SCFA content were similar in case and control horses, and were within the range for healthy horses, horses with FFL were not considered to suffer from hindgut acidosis or osmotic diarrhoea.

### Faecal physical characteristics

Faecal DM, particle size distribution and volume of free liquid was similar between case and control horses. Faecal DM content and volume of free liquid however only show the quantity of water in faeces, and not how the water is distributed or held in the faecal matrix. Water holding capacity can show how much liquid that can be absorbed by faecal particles and could therefore be of interest in FFL. Faeces from case compared to control horses had lower water holding capacity. Faecal water holding capacity may be related to the type and amount of fibre present in the faeces, due to the variation in hydrophilic properties in different fibre fractions in the digesta [24–26]. The amount and type of fibre present in the digesta and faeces may vary in horses fed the same diets, as it have been reported previously that fibre digestibility differs with individual in horses [27]. This variability may result in some fibres being digested to a smaller or larger extent than others in some horses, which could in turn have an impact on the hydrophilic properties of the digesta and/or faeces. In future studies of horses with FFL, individual digestibility of different feeds and fibres should therefore be considered as a factor worth to include.

Particle size distribution in faeces is normally considered an indicator of the function of mastication in equines [11], as there is no further reduction of digesta particle size during digesta passage through the rest of the gastrointestinal tract [28]. Faecal particle size distribution was similar in case and control horses in the present study, indicating that mastication problems were not a cause of FFL. It has previously been reported that variation in digesta particle size can be large between individual horses [29]. However, digesta particle size has also been reported to be affected by feed type, being larger for horses fed hay compared with horses fed hay plus pelleted concentrates [11]. Although similar particle size distribution was observed in faeces from case and control horses in the present study, those in sub-study I had a higher proportion of smaller particles (< 1 mm) than those in sub-study II. The reasons for the different findings remain open.

No difference in the volume of faecal sand were shown between case and control horses in the present study. Presence of sand in equine faeces has previously been associated with sand accumulation in the hindgut, which may result in disturbances such as diarrhoea and impaction colic [12]. However, presence of sand in faeces is not a fully reliable indicator of sand accumulation in the hindgut [30], radiography of the abdomen is recommended for diagnosis [31]. Sand accumulation in the hindgut could therefore not be ruled out as a contributing factor in the etiology of FFL in horses, and in future studies it would be beneficial to include it as a factor to investigate further.

### Limitations of the study

Limitations of the study include variation in methods for sampling of faeces. In sub-study I, samples were collected by the horse-owner from fresh faeces, while in sub-study II samples were collected from the rectum by a veterinarian. Despite providing careful instructions for sampling to horse owners in sub-study I, differences in sampling could have resulted in differences in both chemical and physical variables depending on how they were handled at sampling. This may have led to over- or underestimation of variables such as the amount of liquid and sand.

## Conclusions

To the authors’ knowledge, this is the first study to investigate faecal chemical composition and physical characteristics in horses with FFL. The results showed that case horses had lower concentration and proportion (of total SCFA) of lactic acid and lower water holding capacity of faeces compared to control horses. Case and control horses had similar total SCFA, pH and osmolality, indicating that hindgut fermentation was similar. Particle size distribution, dry matter content and volume of sand in faeces were similar in case and control horses, indicating that these variables were not associated with FFL. The differences observed between case and control horses in faecal lactic acid concentration and proportion and water holding capacity are of interest in further studies of horses with FFL.

## Materials and methods

### General

The study comprised two sub-studies with one pair of case (with FFL) and control (without FFL) horses on each farm. Data from the two sub-studies were treated separately, due to differences in methods used during collection and analysis of samples. In sub-study I, case and control horses were located in Sweden and Norway. In sub-study II, case and control horses were located in Germany. The definition of a case horse was a horse showing two-phase characteristics of faeces (one solid and one liquid phase), and the definition of a control horse was a horse showing only a solid phase and no separate liquid phase of their faeces. Inclusion criteria for all horses in the study comprised no change in feeds or feeding, no change of stable, no signs of pyrexia, and no ongoing medical treatment during the preceding 6 months, and the horse had to be at least 2 years old. Additional requirements for control horses included no clinical signs of any gastrointestinal tract problems during the preceding 6 months. Further information about the horses in the study can be found in Lindroth et al. [32] Owners had to provide results from analysis of faecal egg counts (FEC) and information on the use of anthelmintic drugs prior to being included in the study. No horse in the study had FEC values exceeding 100 EPG. All horse owners signed a written informed consent before being accepted as participants in the study.

### Sub-study I (Sweden-Norway)

A total of 100 horses (50 horse pairs of horses containing one case and one control horse on the same farm) were recruited to sub-study I through an advertisement via internet communication channels connected to the Department of Animal Nutrition and Management, Swedish University of Agricultural Sciences (SLU). The farms were located in Norway (*n* = 20) and Sweden (*n* = 30). The horses in each pair were kept in the same stable or loose housing system, fed the same forage and kept in the same or adjacent paddocks. Horses were mainly of warmblood type, geldings and used for leisure riding, and on average aged 13 ± 5.7 (case) and 10 ± 5.3 (control) years. Case horses were on average fed 89% roughage and 11% concentrates in their total daily feed ration, and control horses on average 90% roughage and 10% concentrates (full details of horses, feeding and management available in Lindroth et al. [32, 33]).

#### Sample collection

Non-invasive methods were used for collection of faecal samples. Horse owners were asked to provide a faecal samples from their horses during three SP (SP): October/November 2016 (SP1), December 2016/January 2017 (SP2) and February/March 2017 (SP3). Case and control horses on each farm were sampled on the same day in all SPs. A sampling kit with illustrated instructions and materials for collection of faecal samples was provided to all participants prior to each of the three SPs. Instructions included collection of approximately 250 g faeces from the ground or stable floor immediately after defecation and placing samples in double plastic bags that were closed immediately. The horse owners were instructed to sample only a part of the faeces that had not touched the ground or floor, to use new disposable clean gloves for each horse, and to carefully close the bags and send them directly by postal service to the laboratory at the Department of Animal Nutrition and Management, SLU, Uppsala. If samples were not sent directly after collection horse owners were instructed to keep the samples in a fridge (4–5 °C) until samples were sent by post. Samples were discarded if the time from sampling to arrival at the laboratory exceeded 4 days, and horse owners were then asked to send new samples within the current SP.

#### Analysis of faecal chemical composition

On arrival at the laboratory, faecal fluid was obtained by pressing faecal samples with a handheld fruit press. The pH value was measured immediately in the faecal fluid, using a pH meter fitted with a glass electrode (WTW pH 315i, Weilheim, Germany). After pH measurement, the faecal fluid was kept at − 20 °C until analysis of SCFA. Concentrations of SCFA (including acetic, propionic, *i*- and *n*-butyric, *i* and *n*-valeric and lactic acid) were analysed by high performance liquid chromatography according to Andersson & Hedlund [34]. The DM content was determined by drying faeces samples in two steps. First, samples were dried for 18 h at 55 °C and, after air equilibration, weighed and ground in a hammer mill to pass a 1.0-mm screen. Samples were then dried again for 20 h at 103 °C. Ash content was determined by incineration for 3 h at 550 °C. The content of sodium (Na), potassium (K), calcium (Ca), phosphorus (P), magnesium (Mg) and sulphur (S) were analysed by plasma emission spectroscopy (Spectro Analytical Instruments GmbH & Co., Kleve, Germany), on samples extracted with HNO_3_ according to Bahlsberg-Pålsson [35]. Values below the lower detection level (0.1 g/kg sample) were transformed to half the lower detection level (0.05 g/kg) before statistical analysis. Samples of faecal liquid used for pH and SCFA analysis were centrifuged at 16000×g for 5 min and the supernatant was used for measurement of osmolality (Advanced Osmometer, Model 3250, Advanced Instruments Inc., Norwood, MA, USA). The buzzpoint was kept at 2000 to avoid premature freezing of samples. Calibration of the osmometer was performed after every 30 sample, using calibration liquids (Clinitrol 290 Reference solution and Calibration Standard, Advanced Instruments, INC., Norwood, MA) and distilled water.

#### Analysis of physical characteristics of faeces

Water holding capacity (mass of water absorbed per mass sample) of faeces was measured according to Gardner [36], modified as follows: 5 mL of dried and milled (as described previously) faeces were placed in a graded 50 mL test tube and the weight of the sample was recorded. Distilled water was added to the tubes in an amount corresponding to 10 times the weight of the faecal sample and the mixture was left at room temperature (20–22 °C) for 24 h to sediment. After 24 h, three different phases were visible in the test tubes, a solid phase with small particles at the bottom, a liquid phase in the middle and a solid phase with large particles at the top. The volume (mL) of each of the three phases was recorded. The volume of absorbed water was calculated by subtraction of the middle liquid phase in the test tube from the volume of distilled water added to the tube. The water holding capacity of the faeces was then calculated as mL absorbed water per g dried faeces. Volume of faecal liquid and of sand in faeces samples was determined by centrifugation, where samples of 10 mL faecal liquid used for pH and SCFA analysis were centrifuged (HERMLE, Z383K, Skafte Medlab, Mölndal, Sweden) for 1 min at 1000 rpm. Radius of the centrifuge was set at 0.5, brake at 9.0 and pre-cool at 10.0. The total volume of fluid (mL) in test tubes was recorded by subtracting the volume of the solid phase in the bottom of the test tubes from the total sample volume. The solid phase in the bottom of test tubes consisted of sand particles and this volume was recorded as mL sand. Volume readings were made with 0.01 ml accuracy.

### Sub-study II (Germany)

A total of 64 horses (32 horse pairs of case and control horses) were recruited to sub-study II from the clientele of an equine clinic (Pferdeklinik An der Rennbahn, Iffezheim, Germany), where all farms were located in southern Germany (*n* = 32). Information on feeding, medical treatments and anthelminthic practices in the 3 months before the start of the study was collected for all horses through an on-site interview with owners, conducted using a standardised protocol. Horses were mainly of warmblood type, geldings and used for leisure riding, and had an average age of 15 ± 7.1 (case) and 12 ± 6.5 (control) years. Case horses were fed diets consisting of on average 73% roughage and 27% concentrates, and control horses were fed on average 74% roughage and 26% concentrates.

#### Sample collection

Faecal samples were collected once from all horses by rectal sampling performed by the same veterinarian on all farms. Case and control horses on each farm were sampled on the same day. Faecal samples were stored within 2 h from collection at 4 °C until analysis. On the sample collection day, all horses underwent a clinical health check performed by the sampling veterinarian. Horse body weight was determined using a transportable scale.

#### Analysis of faecal chemical composition

Faecal pH was analysed by use of pH meter (pH Meter Piccolo, Hanna, Kiel, Germany). Concentrations of SCFA (including acetic, propionic, i- and n-butyric, i and n-valeric and caproic acid) in faecal samples were analysed by gas chromatography (GC 14 A, Shimadzu, Duisburg, Germany). D- and L-lactic acid concentration was analysed by an UV method using lactate dehydrogenase in combination with a commercial test kit (Boehringer, Ingelheim, Germany). Dry matter content in faeces was determined after oven drying (103 °C) to constant mass. Crude ash content was obtained by incineration of the samples for 6 h at 600 °C. Faecal content of K were analysed by flame photometry and of Mg by atomic absorption spectrophotometry. Values below the lower detection level (0.1 g/kg sample) were transformed to half the lower detection level (0.05 g/kg) before statistical analysis.

### Analysis of faecal particle size distribution in sub-studies I and II

Faecal samples from sub-studies I and II were analysed for particle size distribution. Samples from sub-study I were shipped to the laboratory in Germany in order to use the same standardized method used in sub-study II. A subsample of 15 g faeces was soaked in 500 mL distilled water, mixed and equilibrated in a fridge (+ 4 °C) for 12 h. Sieves (8 mm, 4 mm, 2 mm and 1 mm mesh size) were placed on top of each other, with the smallest diameter sieve at the bottom, creating a sieve tower that was placed in a bucket with a valve at the bottom. The mixture of faeces and distilled water was poured slowly onto the upper sieve and the valve was opened to induce a constant slow water flow. When the mixture had run through all sieves, the particles remaining on each sieve were collected on petri-dishes, one for each mesh size. The petri dishes containing the particles were dried for 18 h at 60 °C, cooled and weighed. The petri dishes were weighed separately before (tare weight) and after drying. Particle size distribution was calculated as dry weight of sample on each sieve as a percentage of dry weight of the total subsample. The fraction of the material washed through the sieve with the finest mesh size was calculated from the dry weight of the total subsample minus the sum of dry weight on the four sieve fractions.

### Data treatment

Total SCFA was calculated as the sum of acetic, propionic, *i-* and *n-*butyric, *i*- and *n*- valeric and D- and L-lactic acid concentrations. Proportion of individual SCFAs to total SCFA was calculated and expressed as a percentage. The ratio (C2 + C4)/C3 was calculated from the sum of acetic (C2) and i- and n-butyric acid (C4) concentrations divided by propionic acid (C3) concentration.

### Calculations and statistical analysis

All statistical analyses were performed using SAS version 9.4 for Windows (Statistical Analysis System Institute Inc., Cary, NC, USA). Mean and standard deviation were calculated and presented in tables. Boxplots were used to illustrate data range. In sub-study I, comparisons between case and control horses were performed using the generalised linear mixed model (PROC GLIMMIX) procedure, with effect of SP and interactions between case/control and SPs included as fixed effects, and with repeated measurements on horses and farm (ID) included as random effects:

*Y*_*ijkl*_ = *μ* + (case/control)_*i*_ + (SP)_*j*_ + (case/control⁎SP)_*ij*_ + (id⁎case/control)_*ik*_ + (error)_*ijkl*_ (id*case/control)_*ik*_ + (error)_*ijkl*_*.*

where the term “error” is the random residual with mean = 0 and variance σ2.

In sub-study II, comparisons between case and control horses were performed using the generalised linear mixed model (PROC GLIMMIX) procedure with farm (ID) included as random effects:

*Y*_*ijkl*_ = *μ* + (case/control)_*i*_ + (id⁎case/control)_*j*_ + (error)_*ij*_*.*

where the term “error” is the random residual with mean = 0 and variance σ2.

For analysis of faecal particle size, including data from sub-study I and II, comparisons between case and control horses were performed using the generalised linear mixed model (PROC GLIMMIX) procedure with effect of study and interactions between case/control and study included as fixed effects, and with repeated measurements on horses and farm (ID) included as random effects:

*Y*_*ijkl*_ = *μ* + (case/control)_*i*_ + (sub-study)_*j*_ + (case/control⁎sub-study)_*ij*_ + (id⁎case/control)_*ik*_ + (error)_*ijkl*_*.*

where the term ‘error’ is the random residual with mean = 0 and variance σ^2^.

Missing values were treated as such in statistical analysis. Differences at *p* ≤ 0.05 were regarded as statistically different.

## Data Availability

The datasets used and/or analysed during the current study available from the corresponding author on reasonable request.

## Abbreviations

C2 + C4/C3: Ratio between sum of acetic (C2) and butyric acid (C4) to propionic acid (C3)
FFL: Free faecal liquid
FWS: Faecal water syndrome
SCFA: Short chain fatty acids
SP: Sampling period
VFA: Volatile fatty acids
